# Tuning methane decomposition on stepped Ni surface: The role of subsurface atoms in catalyst design

**DOI:** 10.1038/s41598-017-14050-3

**Published:** 2017-10-25

**Authors:** Ryan Lacdao Arevalo, Susan Meñez Aspera, Mary Clare Sison Escaño, Hiroshi Nakanishi, Hideaki Kasai

**Affiliations:** 1 National Institute of Technology, Akashi College, 679-3 Nishioka, Uozumi, Akashi, Hyogo, 674-8501 Japan; 2 Department of Applied Physics, University of Fukui, 3-9-1 Bunkyo, Fukui, 910-8507 Japan; 3 Graduate School of Engineering, Osaka University, 2-1 Yamadaoka, Suita, Osaka, 565-0871 Japan; 4 Institute of Industrial Science, The University of Tokyo, Meguro, Tokyo, 153-8505 Japan; 5 Osaka University, 1-1 Yamadaoka, Suita, Osaka, 565-0871 Japan

## Abstract

The decomposition of methane (CH_4_) is a catalytically important reaction in the production of syngas that is used to make a wide spectrum of hydrocarbons and alcohols, and a principal carbon deposition pathway in methane reforming. Literatures suggest that stepped Ni surface is uniquely selective toward methane decomposition to atomic C, contrary to other catalysts that favor the CH fragment. In this paper, we used dispersion-corrected density functional theory-based first principles calculations to identify the electronic factors that govern this interesting property of stepped Ni surface. We found that the adsorption of atomic C on this surface is uniquely characterized by a 5–coordinated bonding of C with Ni atoms from both the surface and subsurface layers. Comparison with Ru surface indicates the importance of the subsurface atoms of stepped Ni surface on its selectivity toward methane decomposition to atomic C. Interestingly, we found that substituting these subsurface atoms with other elements can dramatically change the reaction mechanism of methane decomposition, suggesting a new approach to catalyst design for hydrocarbon reforming applications.

## Introduction

The production of synthesis gas through methane reforming has gained significant research attention in the past years due to its economic and industrial importance. Ru and Ni surfaces are widely used catalysts for this reaction because of their high activity^1,2^. Practically, Ni is more commonly used because of its low cost. However, its efficiency is hindered by its high activity for carbon deposition/formation that deactivates the catalyst^3,4^.

One principal pathway for such carbon formation reaction is the decomposition of methane to atomic carbon^4^. This reaction has been studied extensively both experimentally and theoretically. Ciobica *et al*. studied the decomposition of methane on flat (i.e., (111) terrace site) Ru surface and found that the dissociation path toward CH + 3H is most preferred^1,5^. This was similarly observed by Bengaard *et al*. for the case of flat Ni surface^2^. However, they further noted that the dissociation toward C + 4H is more preferred on stepped Ni surface, suggesting it as a possible nucleation site for carbon. They argued that as carbon atoms adsorb at the step sites, a graphene layer can form and nucleate into an island that may continue to grow forming pyrolytic, encapsulating, or whisker carbon, as observed in electron microscopy^3^. This picture of carbon formation on stepped Ni surface is verified by *in situ* electron microscopy of the initial carbon formation as reported by the Norskov group^6^.

Recently, we reported the decomposition of methane on the stepped site of Ru surface^7^. We found that the most preferred decomposition path on this surface is the formation of CH + 3H fragments, similar to the cases of flat surfaces of Ru and Ni mentioned earlier. This indicates that stepped Ni surface is uniquely selective toward the decomposition pathway of methane to C + 4H. However, the electronic properties that govern this interesting property of stepped Ni surface has never been clarified. This is imperative as it is by now clear that the atomic-scale electronic interaction of molecules and catalysts can serve as basis for the design of catalytic materials. In this paper that utilized dispersion-corrected density functional theory (DFT) – based first principles calculations, we sought to identify the electronic origin of the high activity of stepped Ni surface toward carbon formation by comparing it to the case of stepped Ru surface. From our results, we developed new insights into the modification of Ni catalyst that can weaken the high stability of atomic carbon on the surface and thereby change the thermodynamics of methane decomposition pathways.

## Results and Discussion

This section is divided into three parts. First, we calculated the thermodynamics of methane decomposition pathways on Ru and Ni surfaces to confirm the earlier reported DFT results. Then, we explain the electronic properties that govern these decomposition pathways. Finally, we discuss new insights for catalyst design.

### Revisiting methane decomposition pathways

**Figure 1:**
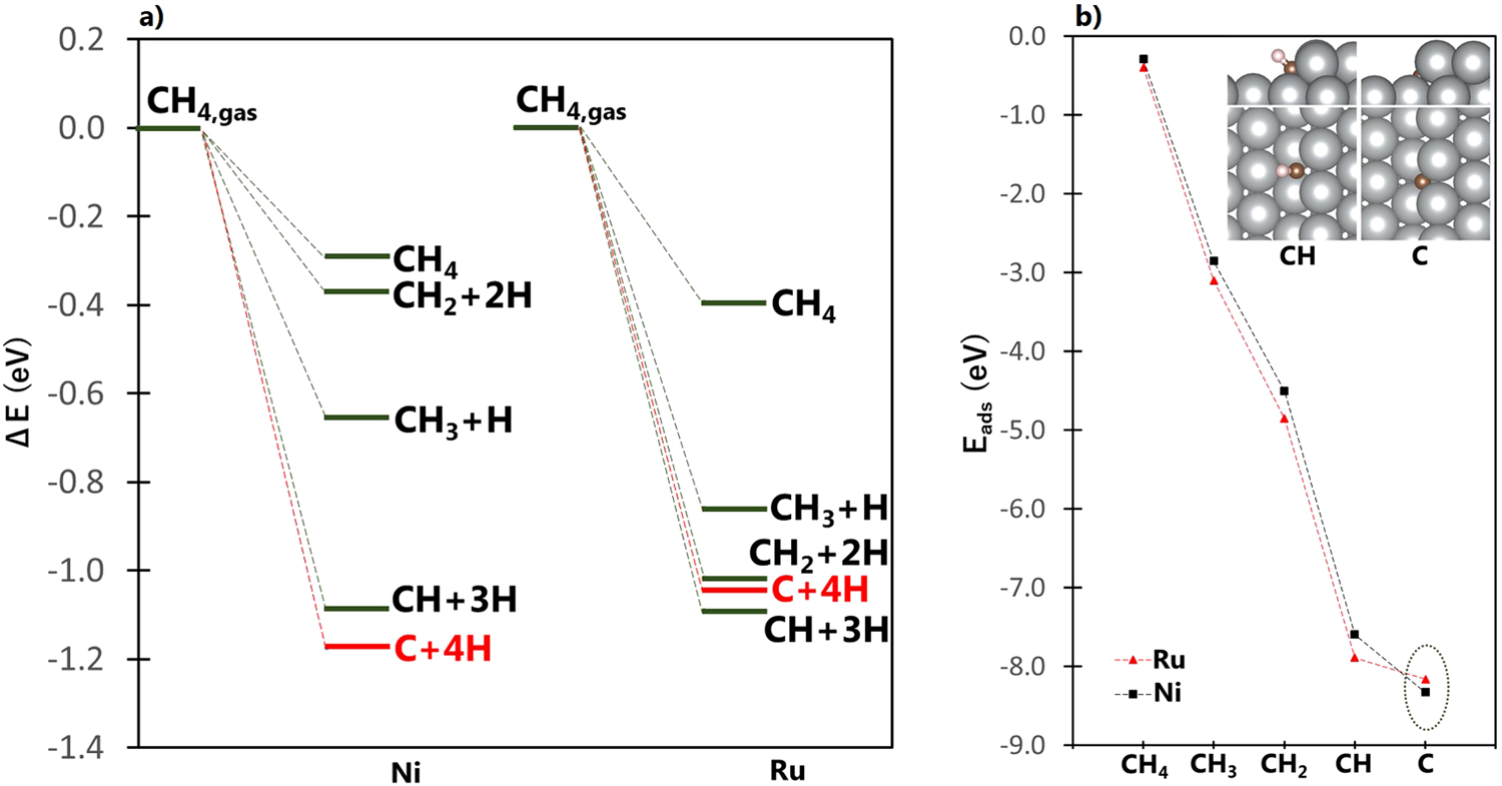
(**a**) Reaction energy (ΔE) diagram for CH_4_ dissociative (or molecular) adsorption to CH_y_ + zH (y + z = 4) on stepped Ni and Ru surfaces. Similar energy diagrams for CH_3_, CH_2_, and CH can be found in the Supplementary Information. (**b**) Adsorption energy (E_ads_) for CH_x_ (x = 0 to 4) species on Ru and Ni stepped surfaces. The inset figures show the optimal adsorption configurations of CH and C fragments. While the magnitude of adsorption energies is in the order CH_4_ < CH_3_ < CH_2_ < CH < C, this does *not* indicate that the sequential dehydrogenation steps from CH_4_ to C are all exothermic because of the different CH_x_ gas reference states. The exothermicities of different dehydrogenation steps can be deduced only from Fig. 1a, where the number of atoms is conserved for each decomposition path.

Figure 1a shows the reaction energy diagram for CH_4_ molecular/dissociative adsorption on stepped Ni and Ru surfaces. Similar diagrams for the cases of CH_3_, CH_2_, and CH are shown in the Supplementary Information. The reaction energy ΔE, is defined as the difference between the energy of the adsorbed CH_y_ + zH (with y + z = 4) species and the summed energies of isolated CH_4_ and slab. That is,

1 $${\rm{\Delta }}{\rm{E}}={{\rm{E}}}_{{({{\rm{CH}}}_{{\rm{y}}}+{\rm{zH}})}_{{\rm{ads}}}}-({{\rm{E}}}_{{{\rm{CH}}}_{4}}+{{\rm{E}}}_{{\rm{slab}}})$$

It can be noted from Fig. 1a that the dissociative adsorption of CH_4_ favors the formation of C and CH fragments for the cases of stepped Ni and Ru surfaces, respectively. The same trend was also observed for the decomposition of CH_3_, CH_2_, and CH, as reported in the Supplementary Information. This confirms the DFT results mentioned earlier that suggest the stepped site of Ni surface to be the nucleation site for graphene formation^2^. Interestingly, the preference of stepped Ni surface toward C fragment formation is not observed for the case of stepped Ru surface. As shown in Fig. 1a, CH_4_ favorably dissociates toward CH fragment on stepped Ru surface, as similarly noted for the case of flat Ni surface that was mentioned earlier and confirmed by our own calculations (see Supplementary Information). It can be observed that the difference in ΔE for CH+3H and C+4H states are in the order of 50 meV. Considering that these decomposition fragments are composed mainly of small hydrogen atoms, an energy difference of a few meV is significant. To illustrate this, our previous works on hydrogen have shown that an energy difference of as low as 40 meV (3.86 kJ/mol) is significant enough to determine the site preference of this small atom on the surface (e.g., ref.^8^), which is important in studying the diffusion path and quantum states of hydrogen atom on the surface.

It can be seen from Fig. 1a that the most stable products of methane dissociative adsorption are CH and C fragments. As shown in the inset of Fig. 1b, both fragments adsorb at the 5-fold site, as similarly reported by Galea and coworkers^9^. For adsorbed C, the average C–M (M = Ni, Ru) distances are 1.82 Å and 2.03 Å for Ni and Ru surfaces, respectively. For adsorbed CH, the average C–M distances are 1.94 Å and 2.15 Å for Ni and Ru surfaces, respectively. These indicate that the CH and C fragments are both adsorbed at closer distances to Ni atoms on the step site of Ni than for the case of Ru.

To further verify the preference of Ru and Ni surfaces for CH and C as methane decomposition products, respectively, we calculated the activation barrier required to dissociate the CH fragment using climbing-image nudged elastic bond method (CI-NEB)^10,11^. The calculated activation barriers are 1.10 eV and 0.49 eV for Ru and Ni surfaces, respectively, indicating that the thermodynamically preferred CH fragment on Ru surface requires large activation energy for dissociation. These energy barriers compare well with DFT calculations on Ni clusters and close-packed Ru surface^1,5,12–15^. As a rule of thumb, elementary reactions with energy barriers less than ca. 0.8 eV are likely to proceed at room temperature. As a rough estimate of solving the Eyring’s equation, CH dissociation on stepped Ni surface is approximately 10^10^ times faster than on stepped Ru surface at room temperature, and 10^3^ faster at 1000 K.

### Electronic properties

To shed light into the noted preference of stepped Ni surface for the dissociative adsorption of CH_4_ and other CH_x_ (x = 1–3) species toward C fragment, a plot of adsorption energies of CH_x_ (x = 1–4) species on stepped Ni and Ru surfaces is shown in Fig. 1b. Adsorption energy is defined to be the difference in the total energy of the adsorbate-slab system and the summed energies of isolated adsorbate and slab. That is,

2 $${{\rm{E}}}_{{\rm{ads}}}={{\rm{E}}}_{{{\rm{CH}}}_{{\rm{x}},{\rm{ads}}}}-({{\rm{E}}}_{{{\rm{CH}}}_{{\rm{x}}}}+{{\rm{E}}}_{{\rm{slab}}})$$

**Figure 2:**
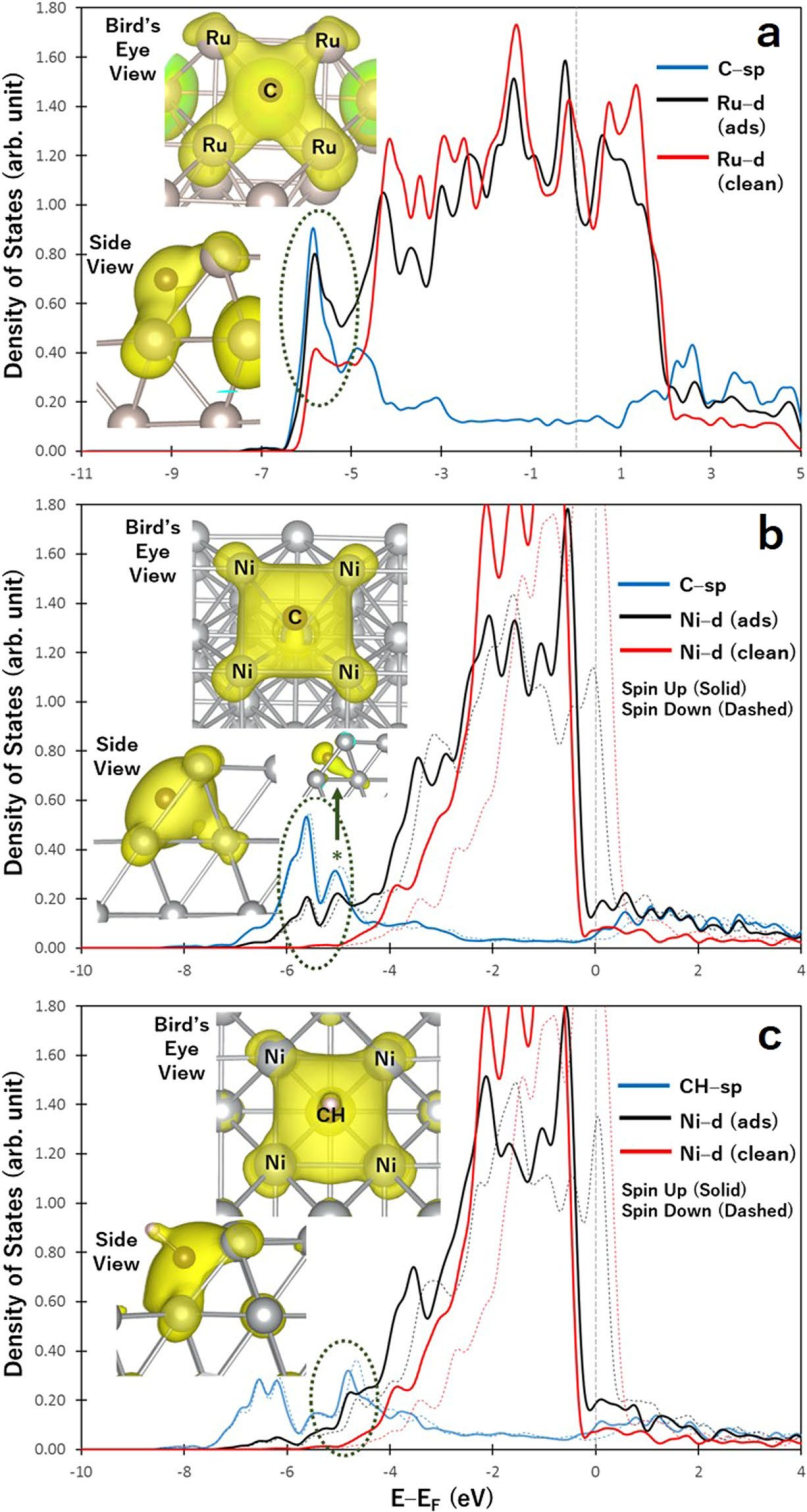
Density of states (DOS) projected on the sp states of C (**a**,**b**) or CH (**c**) and d-band of stepped Ru (**a**), and stepped Ni surfaces (**b**,**c**). “ads” and “clean” denote the surface with and without adsorbate, respectively. The inset figures show the side and bird’s eye views of the partial charge density projected on bonding states within an energy range bound by dashed ellipse. For the case of stepped Ru, the magnetization is completely quenched, making the spin-up and spin-down states to overlap in the figure. The isosurface value for all inset figures is 0.0156787 $e/{a}_{o}^{3}$.

The actual values of adsorption energies, together with the optimal adsorption configurations of CH_y_ + zH (with y + z = 4) species, are reported in the Supplementary Information. The magnitude of adsorption energy is generally lower for Ni surface than Ru surface, except for atomic C which binds stronger on stepped Ni than on stepped Ru surface, as shown by the dashed ellipse in Fig. 1b. The strong stability of atomic C on stepped Ni surface can be explained by the bonding mechanism of C on the surface. Figure 2a and b show the density of states (DOS) projected on the sp state of adsorbed C (plotted as blue curve), and the d band of the metal (Ru or Ni) with (denoted as “ads” and plotted as black curve) and without (denoted as “clean” and plotted as red curve) the adsorbed C. The inset figures show the partial charge density projected on the identified sp–d bonding states at an energy range bound by the dashed ellipse. Based on the inset of Fig. 2a, the delocalization of the electrons around C as it forms bonds with Ru atoms characterize a 4-coordinated C–Ru bonding. The same bonding mechanism (i.e., C bonding with 4 Ru atoms at the surface) was previously reported by our group from our analysis of charge density difference upon the adsorption of C on stepped Ru surface^7^. On the other hand, Fig. 2b shows that for the case of C adsorption on stepped Ni surface, the highly delocalized electrons around C form bonds with five Ni atoms from both the surface and subsurface layers. This 5-coordinated bonding can be achieved since C adsorbs at a closer distance to the step site of Ni than of Ru, as earlier noted. The bonding state, projected at a narrower energy range, that shows the direct interaction between C and Ni atom at the subsurface is denoted by an asterisk in Fig. 2b. This specific bonding state is not observed for the case of Ru surface shown in Fig. 2a. Interestingly, Fig. 2c shows that even though C and CH adsorb at the same 5-fold site on stepped Ni surface, CH only forms bonds with four Ni atoms. This demonstrates the unique bonding mechanism of C on the stepped surface of Ni.

### Ni–M systems (M = 3d, 4d, and 5d transition metals)

Our results and those reported in the literature establish that the step site of Ni surface is highly active for carbon formation because of the high stability of atomic C on this site^2,16^. Several suggested ways to alleviate this problem use various additives such as alkali metal salts, sulfur, boron, and gold, as well as modifying the atomic composition of the step edge^2,17–20^. DFT–based calculations found that additives work by blocking the step sites and hence removing the active sites for carbon formation^2,19^. However, since step sites are more reactive than the close-packed terrace sites for methane activation and most of the elementary steps for the methane reforming process, blocking the step sites is expected to result in the reduction of the reforming rate^2,17–19^.

**Figure 3:**
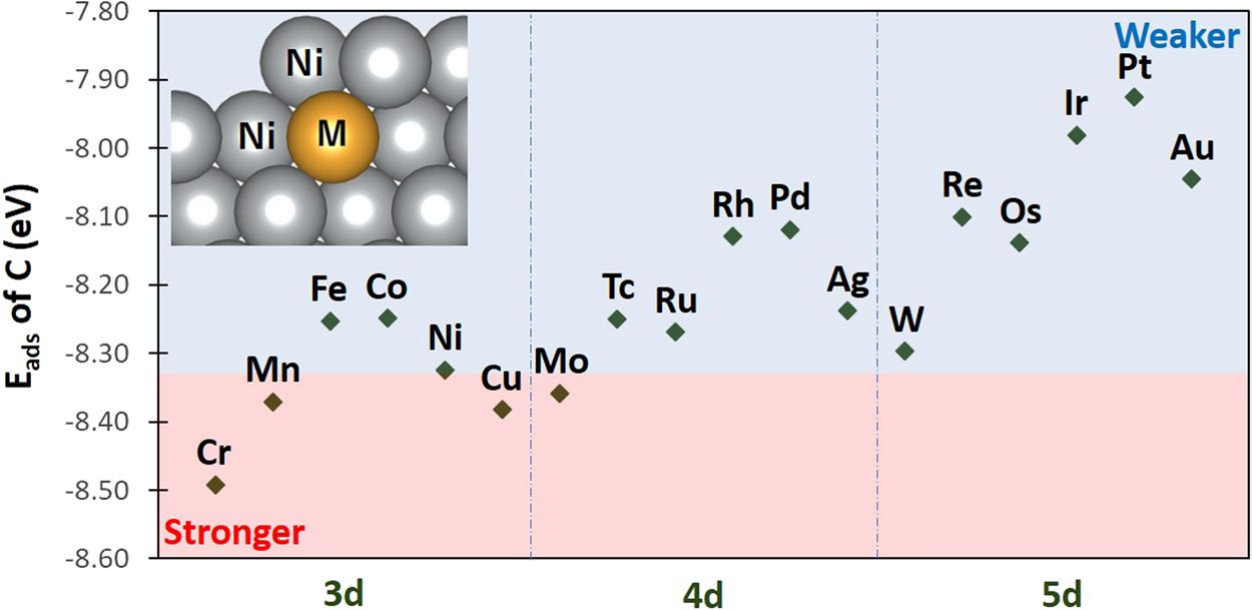
The adsorption energy of atomic C on the stepped surface of Ni–M sytems. The blue and red regions show the M that promote weaker and stronger adsorption of C, respectively, compared to pure Ni.

As reported in the previous section, the high stability of atomic C can be attributed to the 5–coordinated bonding with Ni atoms at the step site of Ni surface. It can be inferred that this binding can be weakened by promoting the 4–coordinated bonding similar to C adsorption on stepped Ru surface. Thus, as an alternative approach to blocking the step sites of Ni surface to prevent carbon formation, it is interesting to determine whether substituting the row of atoms denoted by M in the inset of Fig. 3 with another element will yield the 4-coordinated bonding, thereby weakening the adsorption of atomic C. This modified Ni catalyst will be referred to in this paper as Ni–M system.

**Figure 4:**
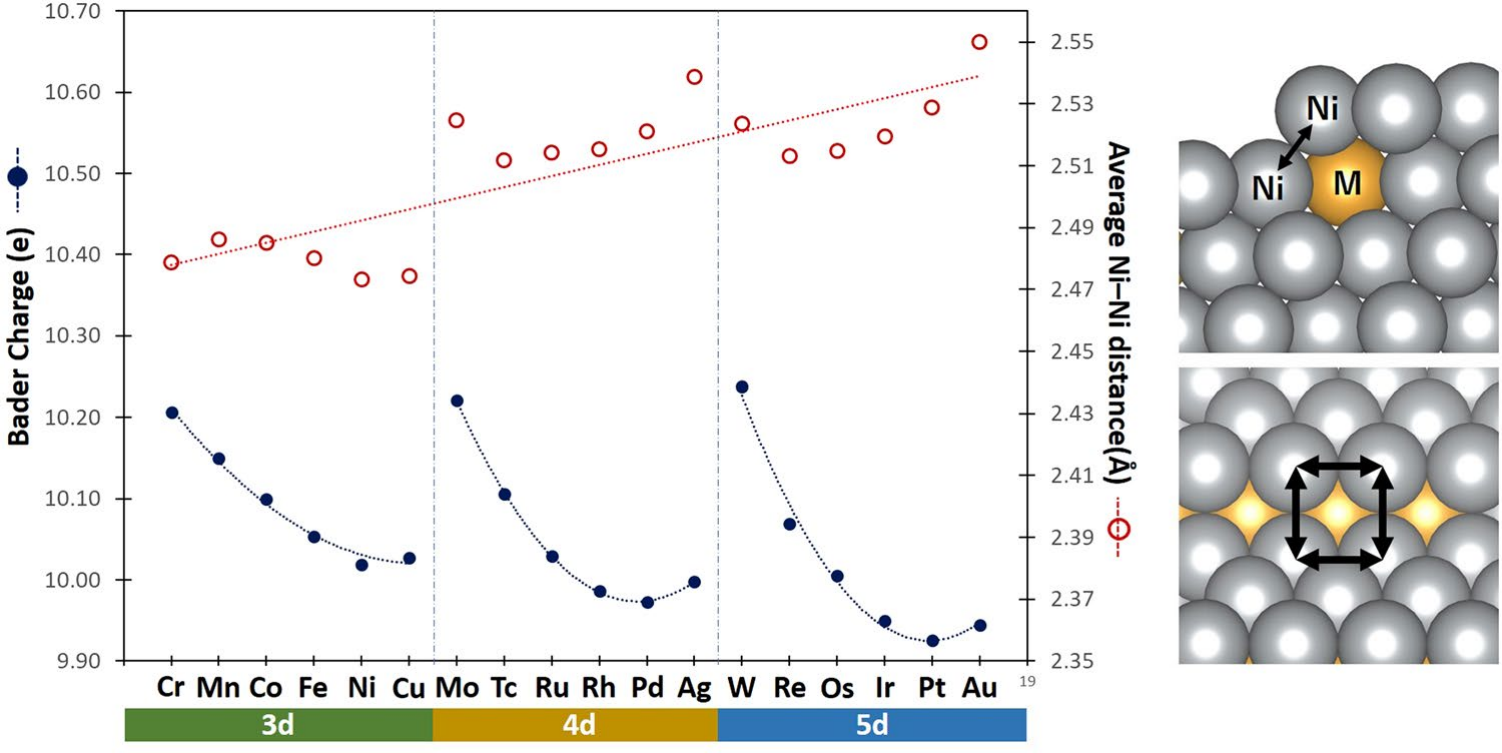
The average Bader charge and average Ni–Ni distance of the four Ni atoms described by the arrows on the figures at the right panel, for the case of clean Ni–M systems. Trend lines are shown to guide the eye.

Figure 3 shows the adsorption energy of atomic C on the stepped surface site of Ni–M system with various M transition metal elements. The figure shows a general trend of decreasing magnitude of adsorption energy in the order of 3d > 4d > 5d elements, with a volcano-shaped behavior for the same row of elements. This observed trend is a complex effect of contributions arising from both ligand and strain factors. Figure 4 shows the average Bader charge and average Ni–Ni distance of Ni atoms at the step site of clean Ni–M surface. The volcano-shaped behavior of Bader charges compares well with C adsorption energies, most pronouncedly for the cases of 4d and 5d metals. It can be inferred from Fig. 4 that the “electron-rich” stepped Ni surface, which is seen markedly for the cases of metals M with near half-filled d orbital (d^4–7^), can facilitate the noted high delocalization of electrons upon C adsorption, resulting in the strong adsorption of C. Additionally, the general trend of decreasing magnitude of adsorption energy in the order 3d > 4d > 5d relates with the increasing average Ni–Ni distance at the step site, suggesting a possible strain effect as the atomic radius of M increases. Upon C adsorption, the step site with longer average Ni–Ni distance promotes longer C–Ni bond length (i.e., farther away from M), thereby weakening its interaction with the subsurface M atom.

**Figure 5:**
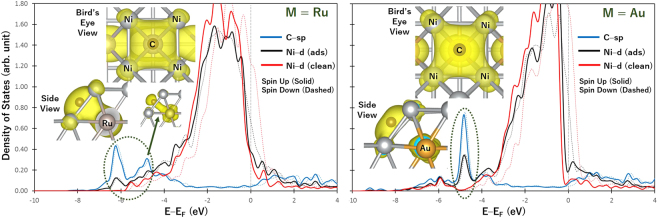
Density of states (DOS) projected on the sp states of C (blue) and d-band of stepped Ni with (“ads” shown in black) and without (“clean” shown in red) the adsorbed C. The inset figures show the partial charge density projected on bonding states within an energy range bound by dashed ellipse.

The density of states for M = Ru and Au cases were analyzed and shown in Fig. 5 to represent the M elements where atomic C has adsorption energies close and far from M = Ni case, respectively. These figures are similar in form with Fig. 2. For the case of M = Ru, the 5–coordinated bonding similar to the case of pure Ni is still seen. The bonding state similar to the one denoted by asterisk in Fig. 2b that indicates the direct interaction of C and subsurface M atom can be seen in the inset of Fig. 5 for M = Ru case. This shows that the small decrease in magnitude of the adsorption energy of atomic C relative to pure Ni is not enough to avoid the 5–coordinated bonding.

Interestingly, Fig. 5 shows that the 4–coordinated bonding is achieved for M = Au case. This is similarly noted for M = Ir and Pt. A sharp peak enclosed by dashed ellipse in Fig. 5 for M = Au case shows the hybridization of C–sp states with Ni–d states at a narrow energy range. An analysis of the d band of Au atom shown in the Supplementary Information indicates that Au atom does not directly interact with C.

**Figure 6:**
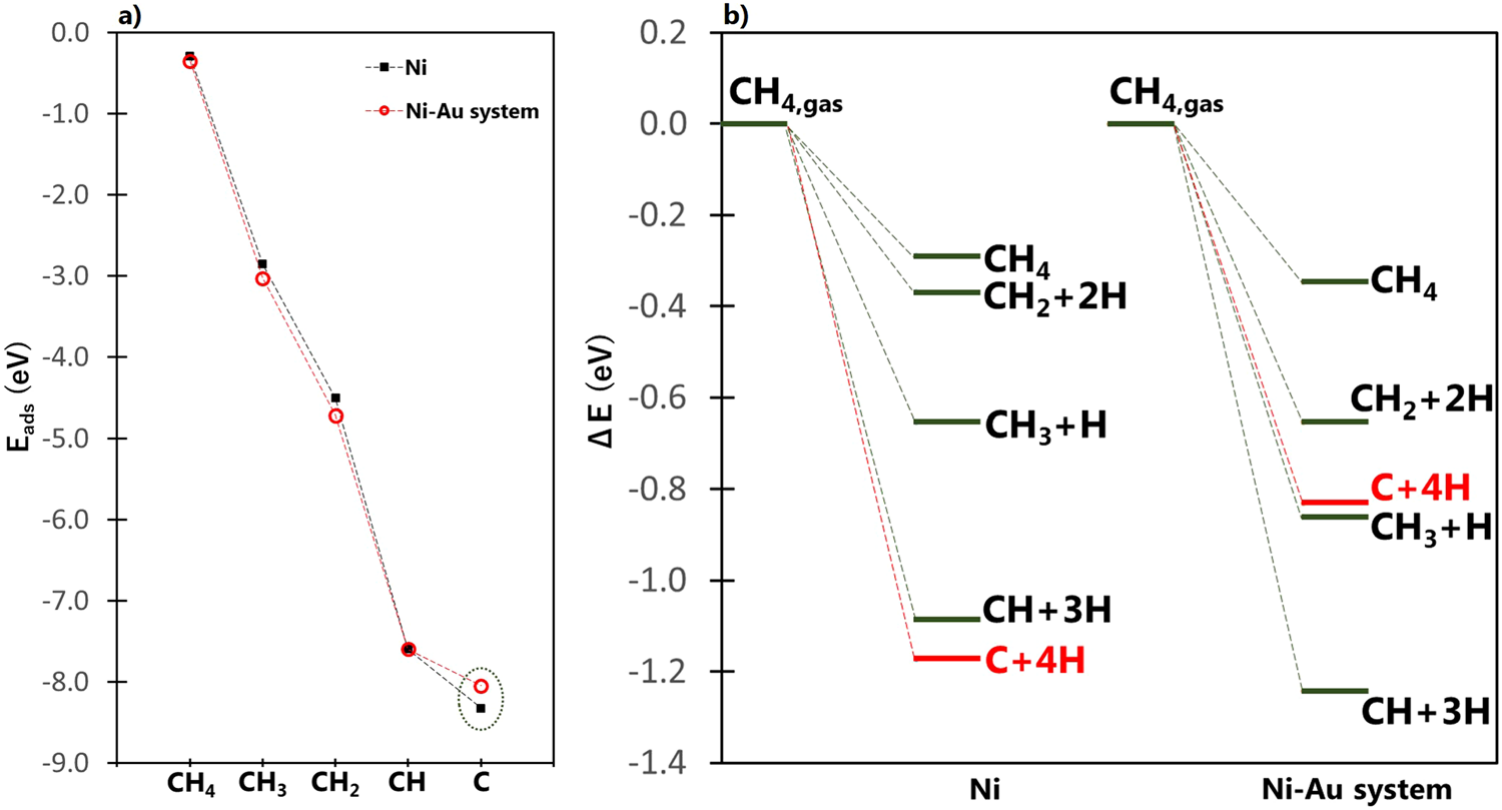
(**a**) The adsorption energy (E_ads_) of CH_x,ads_ (x = 0 to 4) on pure Ni and Ni–Au system. (**b**) Reaction energy (ΔE) for CH_4_ dissociative (or molecular) adsorption to CH_y_ + zH (y + z = 4) on stepped Ni and Ni–Au system.

Figure 6a shows the adsorption energy of methane decomposition fragments on the Ni–Au system, plotted together with the stepped pure Ni surface case. Relative to the pure Ni case, the magnitude of adsorption energies of CH_4_, CH_3_, and CH_2_ on the Ni–Au system have increased by 0.05 eV, 0.17 eV, and 0.21 eV, respectively. On the other hand, the adsorption energy of CH is almost the same for the two systems. As Fig. 6a shows, the decrease in the magnitude of adsorption energy on Ni–Au system can only be seen for the case of atomic C. Its adsorption energy has decreased in magnitude by 0.28 eV. Accordingly, Fig. 6b shows that the Ni–Au system prefers the methane decomposition path toward CH fragment contrary to the preference of pure Ni toward C fragment. Further, it can be noted from Fig. 6b that the reaction energies for the same molecular/dissociative adsorption state of CH_y_ + zH (y + z = 4) species is more exothermic on the Ni–Au system compared to pure Ni, except for the case of atomic C formation.

A number of studies suggested the initial C–H bond dissociation of methane to be a possible rate-determine step for the reforming of methane at the high temperature regime^21–23^. The calculated activation barriers for CH_4_ dehydrogenation to CH_3_ on pure Ni and Ni–Au system are 0.51 eV and 0.33 eV, respectively, suggesting an improved kinetics for this reaction.

It can be reasonably inferred that the weaker binding of C on identified Ni–M systems (such as the Ni–Au system) can make C diffusion and C–C bond formation easier. However, as these catalysts promote the thermodynamic preference toward CH rather than C as methane decomposition fragments, it can be argued that the stability of atomic carbon on the surface is the crucial determining factor for the design of non-coking catalysts.

This particular method of modifying Ni catalyst, as illustrated by the Ni–M systems, features a new approach to catalyst design where subsurface atoms are altered to discriminately tune the adsorption of a particular adsorbate, thereby changing the reaction mechanism happening on the surface. While the aim of our current study is limited to demonstrating the important role of subsurface atoms in the catalysis of methane decomposition to provide insights into the atomic-scale engineering of the step site of Ni surface, it is well recognized that certain metals may segregate on the surface, creating a challenge for the experimental feasibility of subsurface metal substitution for some metal catalysts. For example, the detailed characterization of Ag-Ni alloy catalysts for steam reforming of Wang *et al*. found that Ag atoms form surface alloys and preferentially replace Ni atoms at the step site^24^. Moreover, EXAFS analysis shows that at low concentrations, Au preferentially stays on the surface of Au–Ni alloy and may aggregate with increase in concentration to form small clusters^18^. Nevertheless, Ruban *et al*.^25^ have shown that with the exception of Ag, Pd, and Au, the metals considered in this study have moderate segregation (Pt, Rh, Cu), no segregation (Mn, Ni), and antisegration (the rest of metals) tendencies on Ni host metal. It is hoped that the current study will stimulate further research interests into similar systems, taking into account factors such as surface reconstruction and agglomeration, and geometric and electronic effects, for industrial applications.

## Conclusion

Dispersion-corrected DFT calculations revealed the electronic factors that govern the selectivity of stepped Ni surface toward methane decomposition pathway to a strongly adsorbed atomic carbon. The high stability of carbon on stepped Ni surface is characterized by a unique 5–coordinated C–Ni bonding between atomic carbon at the five-fold site and Ni atoms from both the surface and subsurface layers. Comparison with Ru surface suggests the importance of the subsurface Ni atoms in the adsorption of carbon on the surface. Substituting these subsurface atoms of stepped Ni surface with other elements can discriminately weaken the adsorption of atomic carbon, resulting in the change of reaction mechanism of methane decomposition on stepped Ni surface. These results suggest a new approach to catalyst design aimed at improving the resistance of Ni surface to coke formation in hydrocarbon reforming processes.

### Computational Model

Spin-polarized DFT calculations were carried out using the Vienna ab initio simulation package (VASP)^26–29^ within the generalized gradient approximation based on Perdew-Burke-Ernzerhof (GGA-PBE)^30–33^ functional with an empirical dispersion correction of Grimme (DFT-D2)^34^, as similarly done in our previous works^7,35^. The interaction between ions and electrons was described using the projector augmented wave (PAW) method^36,37^. Plane wave basis sets were employed with an energy cutoff of 400 eV. Electric dipole correction was used to cut the dipole interactions between the repeated image layer systems. The surface Brillouin zone integrations were performed on a grid of 4 × 3 × 1 (for stepped Ru surface), 6 × 6 × 1 (for flat Ni surface used for data in the Supplementary Information), and 5 × 6 × 1 (for stepped Ni surface) Monkhorst-Pack k-points^38^ using Methfessel-Paxton smearing^39^ of σ = 0.2 eV. Conjugate-gradient algorithm^40^ was used to relax the ions into their ground state.

The stepped Ru surface was modeled using four layers of (3 × 4) supercell in the (0001) facet with the top layer having two missing rows, as explained extensively in our previous works^7,35^. On the other hand, Ni(211) surface with 12-atomic layers in a p(1 × 2) slab was used to model the stepped Ni surface. It consists of three-atom-wide terraces with (111) facet structure and one-atom step with (100) character. Vacuum spaces of 14 Å and 10 Å were used to separate the repeated slabs for stepped Ru and Ni surfaces, respectively. The optimization of isolated gas-phase molecules was performed with one free molecule within a 25 × 25 × 25 Å unit cell with full electric dipole moment correction in all directions. The optimal adsorption configuration of the molecules was determined by exhausting a number of different orientations on the surface with the top two layers of the slab fully relaxed in all directions.

## Electronic supplementary material

Supporting Information

## References

[1] Ciobica IM, Frechard F, van Santen RA, Hafner J (2000). A DFT study of transition states for C-H activation on the Ru(0001) surface. J. Phys. Chem. B.

[2] Bengaard HS (2002). Steam reforming and graphite formation on Ni catalysts. J. Catal..

[3] Sehested J (2006). Four challenges for nickel steam-reforming catalysts. Catal. Today.

[4] Wu H (2013). Catalysts.

[5] Ciobica IM, Frechard F, van Santen RA, Kleyn AW, Hafner JA (1999). A theoretical study of CHx chemisorption on the Ru(0001). Chem. Phys. Lett..

[6] Helveg S (2004). Atomic-scale imaging of carbon nanofiber growth. Nature.

[7] Arevalo RL, Aspera SM, Escano MCS, Nakanishi H, Kasai H (2017). First principles study of methane decomposition on B5 step-edge type site of Ru surface. J. Phys.: Condens. Matter.

[8] Ozawa N, Roman TA, Nakanishi H, Kasai H (2007). Potential energy of hydrogen atom motion on Pd(111) surface and in subsurface: A first principles calculation. J. Appl. Phys..

[9] Galea NM, Knapp D, Zieggler T (2007). Density functional theory studies of methane dissociation on anode catalysts in solid-oxide fuel cells: Suggestions for coke reduction. J. Catal..

[10] Henkelman G, Uberuaga BP (2000). A climbing image nudged elastic band method for finding saddle points and minimum energy paths. J. Chem. Phys..

[11] Mills G, Jonsson H, Schenter GK (1995). Reversible work transition state theory: Application to dissociative adsorption of hydrogen. Surf. Sci..

[12] Burghgraef H, Jansen APJ, van Santen RA (1995). Methane activation and dehydrogenation on nickel and cobalt: A computational study. Surf. Sci..

[13] Burghgraef H, Jansen APJ, van Santen RA (1994). Electronic structure calculations and dynamics of methane activation on nickel and cobalt. J. Chem. Phys..

[14] Au C-T, Liao M-S, Ng C-F (1998). A detailed theoretical treatment of the partial oxidation of methane to syngas on transition and coinage metal (M) catalysts (M = Ni, Pd, Pt, Cu). J. Phys. Chem. A.

[15] Au C-T, Ng C-F, Liao M-S (1999). Methane dissociation and syngas formation on Ru, Os, Rh, Ir, Pd, Pt, Cu, Ag, and Au: A theoretical study. J. Catal..

[16] Catapan RC, Oliveira AAM, Chen Y, Vlachos DG (2012). DFT study of the water-gas shift reaction and coke formation on Ni(111) and Ni(211) surfaces. J. Phys. Chem. C.

[17] Besenbacher F (1998). Design of surface alloy catalyst for steam reforming. Science.

[18] Chin YH, King DL, Roh HS, Wang Y, Heald SM (2006). Structure and reactivity investigations on supported bimetallic Au-Ni catalysts used for hydrocarbon steam reforming. J. Catal..

[19] Xu J, Chen LW, Tan KF, Borgna A, Saeys M (2009). Effect of boron on the stability of Ni catalysts during steam methane reforming. J. Catal..

[20] Huang Y, Du J, Ling C, Zhou T, Wang S (2013). Methane dehydrogenation on Au//Ni surface alloys – a first-principles study. Catal. Sci. Technol..

[21] Wei J, Iglesia E (2004). Structural requirements and reaction pathways in methane activation and chemical conversion catalyzed by rhodium. J. Catal..

[22] Nielsen BO, Luntz AC, Holmblad PM, Chorkendorff I (1995). Activated dissociative chemisorption of methane on Ni(100): a direct mechanism under thermal conditions?. Catal. Lett..

[23] Watwe RM, Bengaard HS, Rostrup-Nielsen JR, Dumesic JA, Norskov JK (2000). Theoretical studies of stability and reactivity of CHx species on Ni(111). J. Catal..

[24] Wang H (2017). Steam methane reforming on a Ni-based bimetallic catalyst: Density functional theory and experimental studies of the catalytic consequence of surface alloying of Ni with Ag. Catal. Sci. Technol..

[25] Ruban AV, Skriver HL, Norskov JK (1999). Surface segregation energies in transition-metal alloys. Phys. Rev. B.

[26] Kresse G, Furthmuller J (1996). Efficient iterative schemes for ab initio total-energy calculations using a plane-wave basis set. Phys. Rev. B.

[27] Kresse G, Furthmuller J (1996). Efficiency of ab-inito total energy calculations for metals and semiconductors using a plane-wave basis set. Comput. Mater. Sci..

[28] Kresse G, Hafner J (1993). Ab initio molecular dynamics for liquid metals. Phys. Rev. B.

[29] Kresse G, Hafner J (1994). Ab initio molecular-dynamics simulation of the liquid-metal-amorphous-semiconductor transition in Germanium. Phys. Rev. B.

[30] Perdew. J, Burke K, Ernzerhof M (1996). Generalized gradient approximation made simple. Phys. Rev. Lett..

[31] Perdew J, Burke K, Wang Y (1996). Generalized gradient approximation for the exchange-correlation hole of a many-electron system. Phys. Rev. B.

[32] Becke A (1988). Density-functional exchange-energy approximation with correct asymptotic behavior. Phys. Rev. A.

[33] Lee C, Yang W, Parr R (1988). Development of the Colle-Salvetti correlation-energy formula into a functional of the electron density. Phys. Rev. B.

[34] Grimme S (2004). Accurate description of van der Waals complexes by density functional theory including empirical corrections. J. Comp. Chem..

[35] Arevalo RL, Aspera SM, Escano MCS, Nakanishi H, Kasai H (2017). Ru-catalyzed steam methane reforming: Mechanistic study from first principles calculations. ACS Omega.

[36] Blochl P (1994). Projector augmented-wave method. Phys. Rev. B.

[37] Kresse G, Joubert J (1999). From ultrasoft pseudopotentials to the projector augmented-wave method. Phys. Rev. B.

[38] Monkhorst H, Pack J (1976). Special points for brilliouin-zone integrations. Phys. Rev. B.

[39] Methfessel. M, Paxton A (1989). High-precision sampling for brilliouin-zone integration in metals. Phys. Rev. B.

[40] Stich I, Car R, Parrinello M, Baroni S (1989). Conjugate gradient minimization of the energy functional: A new method for electronic structure calculation. Phys. Rev. B.

