# Supplementary material for: Tuning methane decomposition on stepped Ni surface: The role of subsurface atoms in catalyst design
Source: Sci Rep. 2017 Oct 25;7:13963. doi: 10.1038/s41598-017-14050-3 (PMC5656674; doi:10.1038/s41598-017-14050-3)
Supplement: Supplementary file 1 — Supporting Information [file 41598_2017_14050_MOESM1_ESM.pdf]

Supplementary Information

Tuning methane decomposition on stepped Ni surface: The role of subsurface atoms in catalyst design

Ryan Lacdao Arevalo[a], Susan Meñez Aspera[a], Mary Clare Sison Escaño[b], Hiroshi Nakanishi[a,c,d,*], Hideaki Kasai[a,e]

[a]National Institute of Technology, Akashi College, 679-3 Nishioka, Uozumi, Akashi, Hyogo 674-8501, Japan

[b]Department of Applied Physics, University of Fukui, 3-9-1 Bunkyo, Fukui 910-8507, Japan

[c]Graduate School of Engineering, Osaka University, 2-1 Yamadaoka, Suita, Osaka 565-0871, Japan

[d]Institute of Industrial Science, The University of Tokyo, Meguro, Tokyo 153-8505, Japan

[e]Osaka University, 1-1 Yamadaoka, Suita, Osaka 565-0871, Japan

*Corresponding Author: nakanishi@akashi.ac.jp

Table S1: Adsorption energies in eV of CH_x species (x = 0 to 4).

	Ni [flat]	Ni [step]	Ru [step]
CH_4	–0.258	–0.289	–0.393
CH_3	–2.632	–2.854	–3.099
CH_2	–4.414	–4.505	–4.852
CH	–7.244	–7.594	–7.889
C	–7.299	–8.324	–8.161

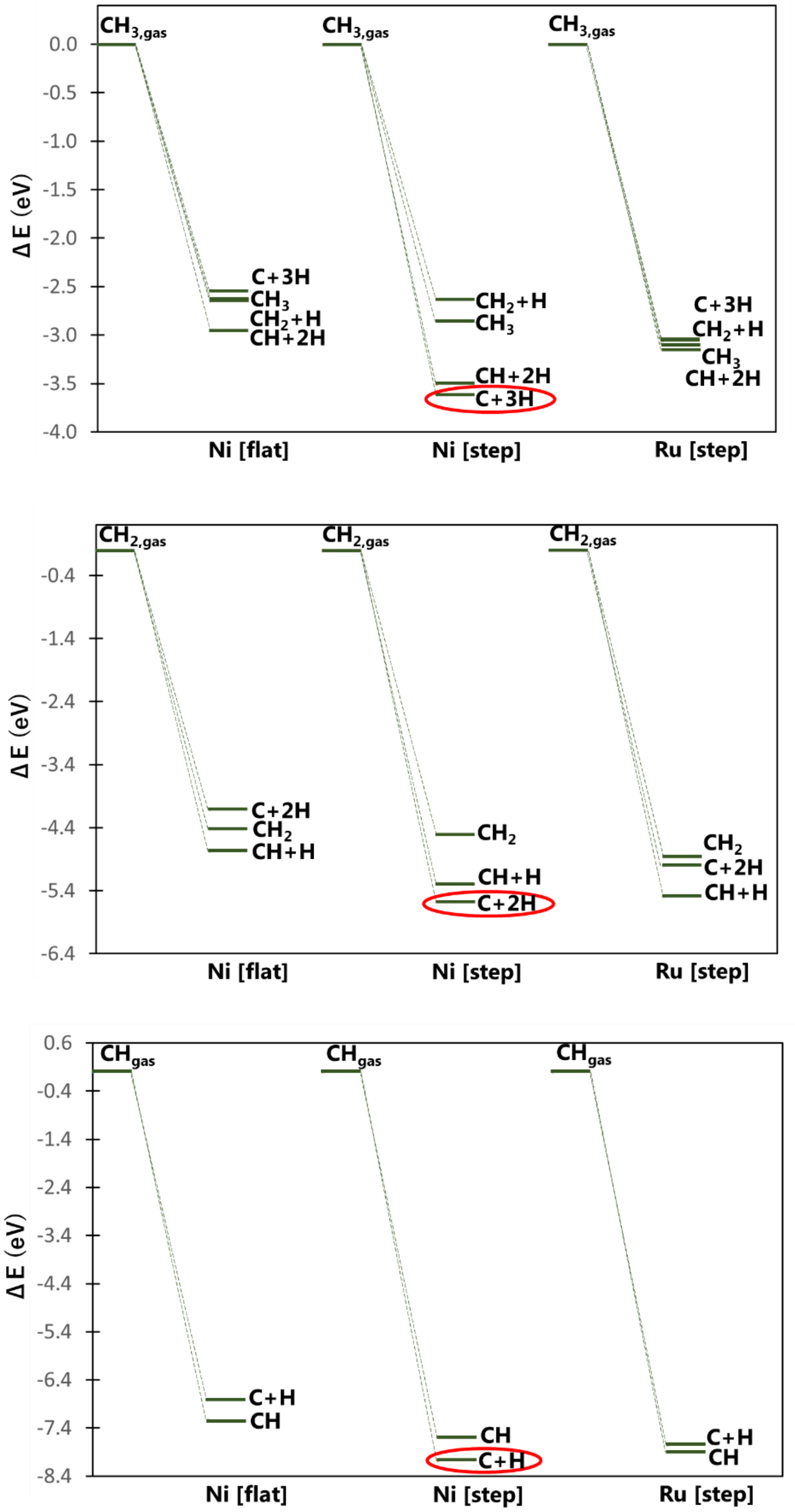

Figure S1: Reaction energy (ΔE) for CH_x (x = 1 to 3) dissociative (or molecular) adsorption on flat Ni, stepped Ni, and stepped Ru surfaces.

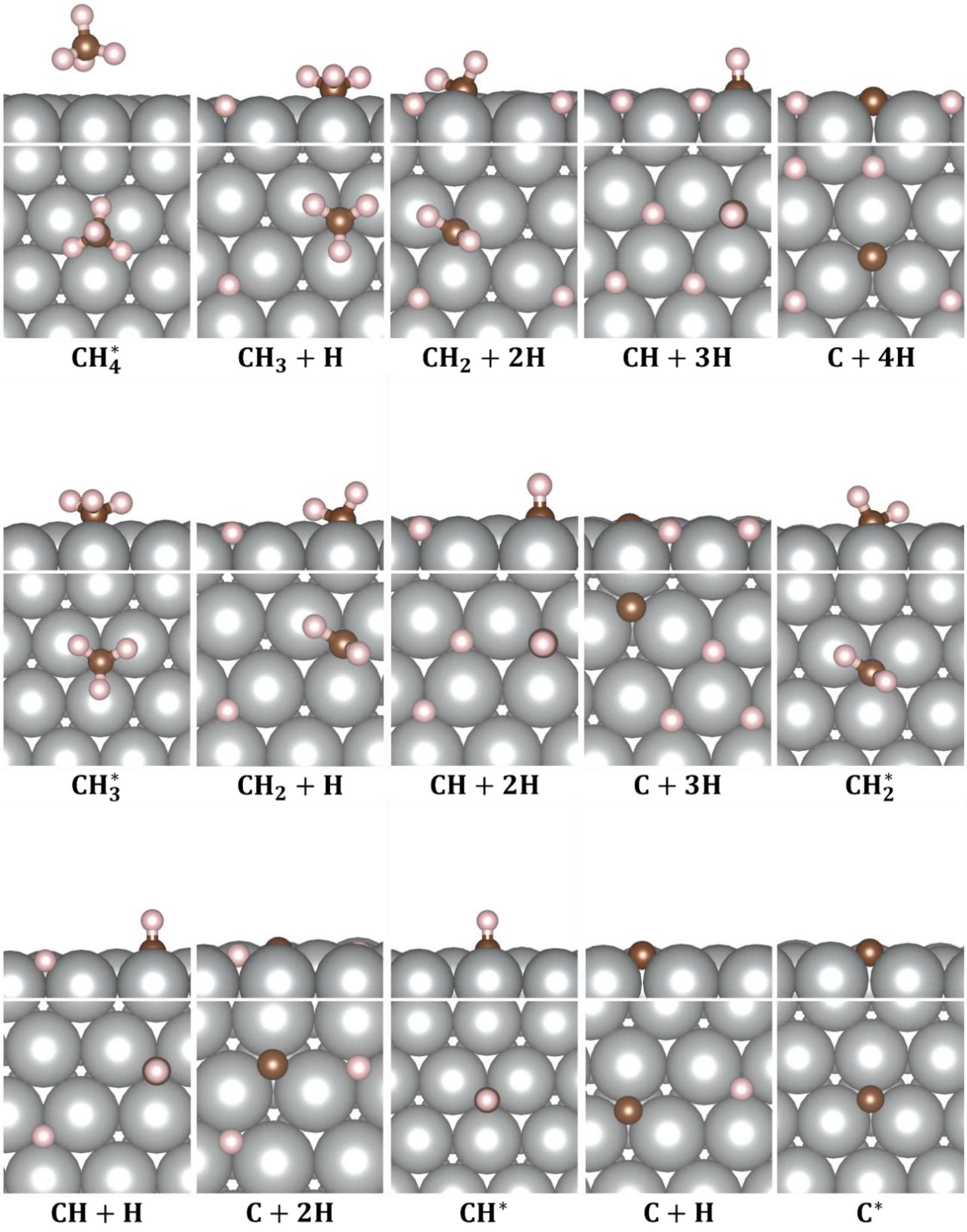

Figure S2: The calculated optimal adsorption configurations of CH_y + zH species on flat Ni surface.

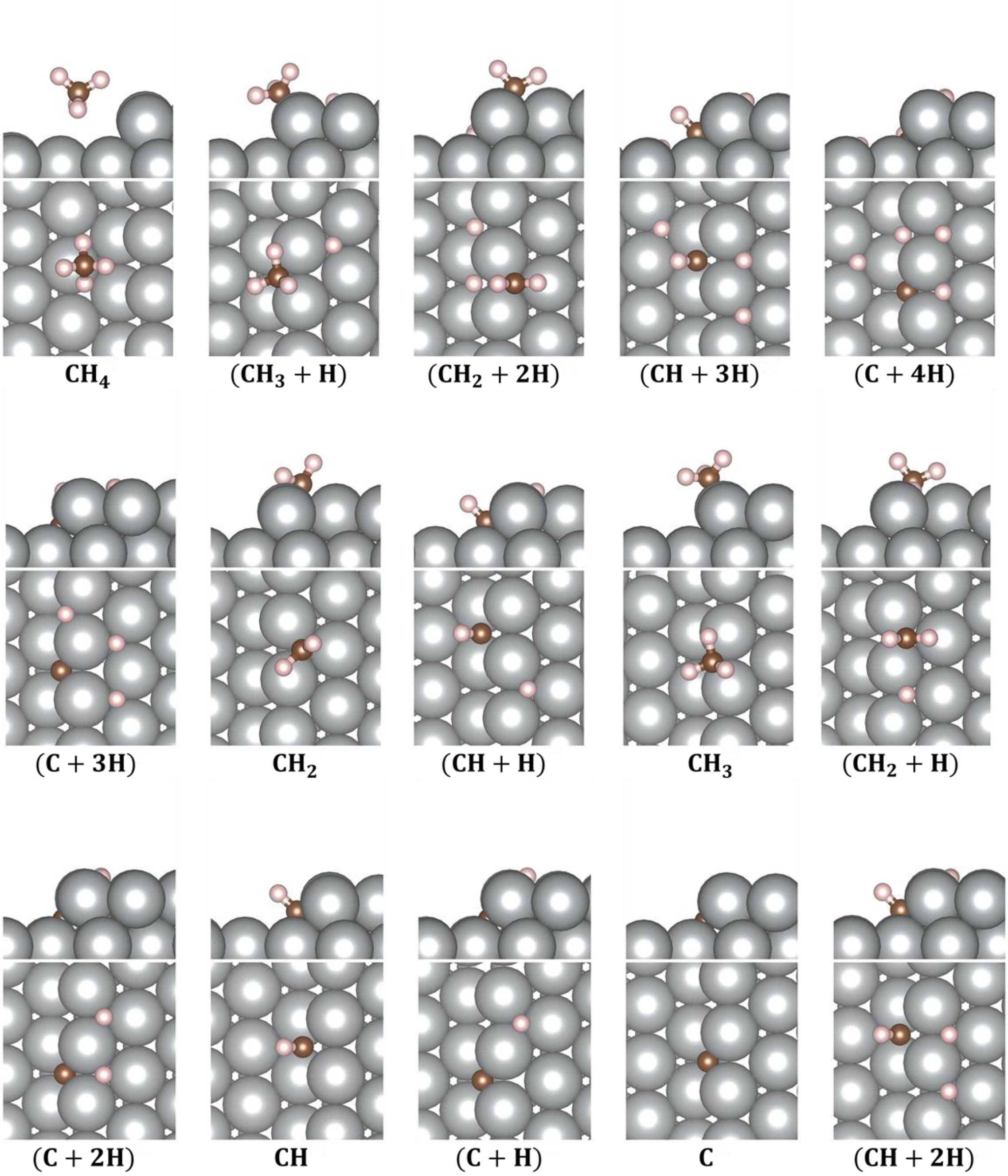

Figure S3: The calculated optimal adsorption configurations of CH_y + zH species on stepped Ni surface.

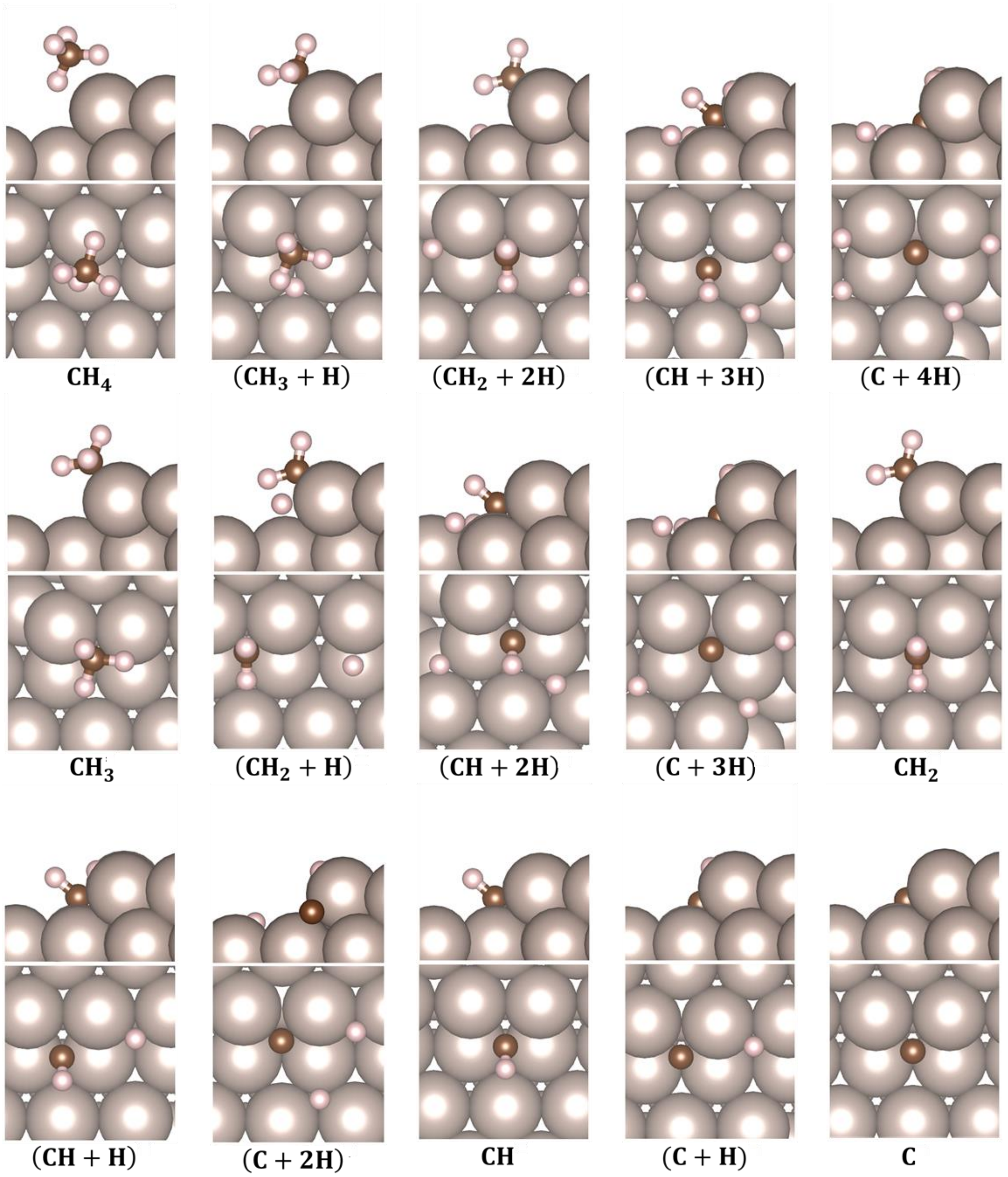

Figure S4: The calculated optimal adsorption configurations of CH_y + zH species on stepped Ru surface.

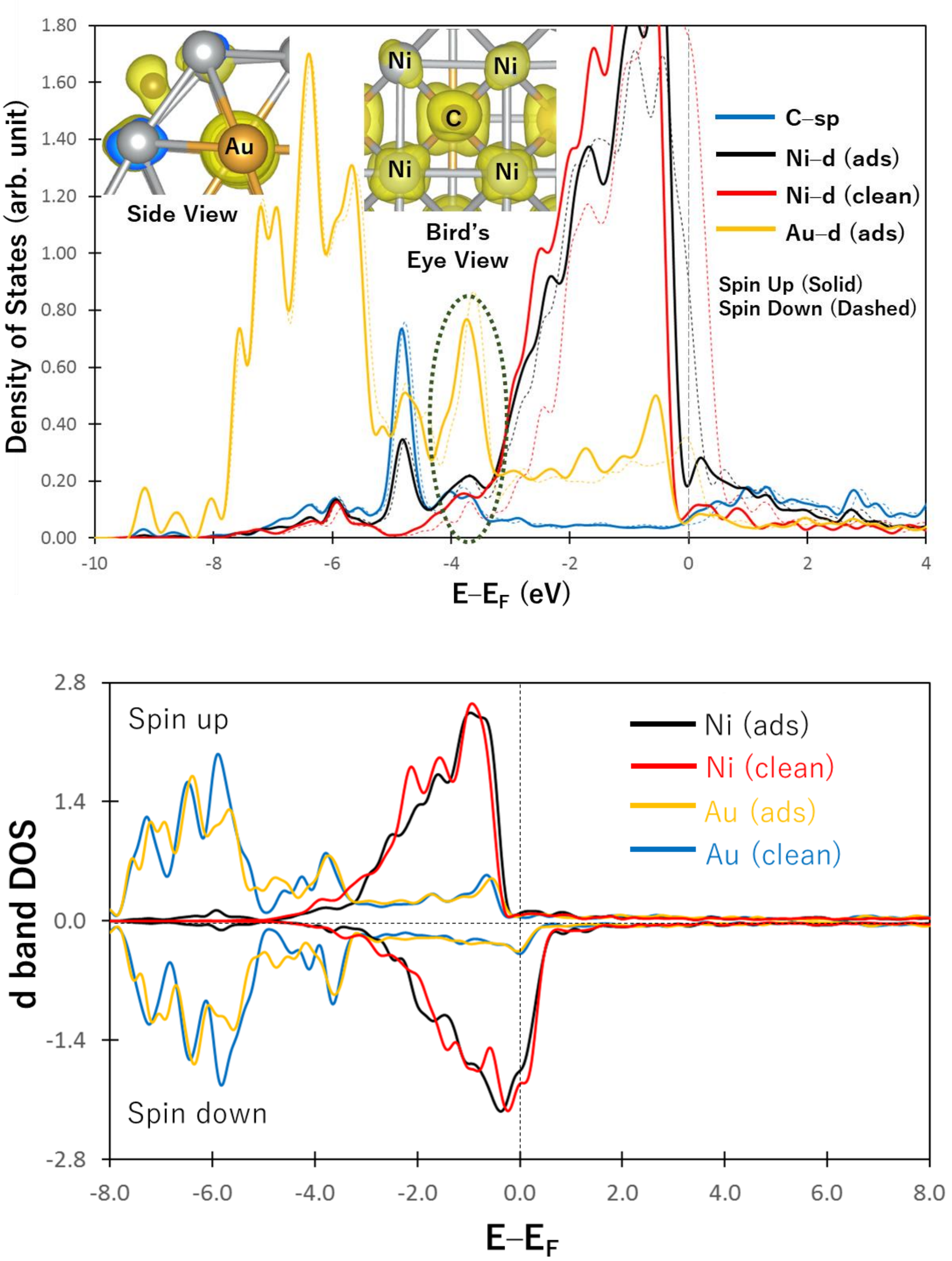

Figure S5: Upper Panel: Density of states projected on the sp states of C (blue), d band of Ni upon C adsorption (black), d band of Ni for clean stepped surface (red), and d ban of Au upon C adsorption (yellow). Lower Panel: d band of Ni and Au atoms with and without the adsorbed C.
